# Supplementary material for: Sensitivity analysis of factors influencing the ecology of mosquitoes involved in the transmission of Rift Valley fever virus
Source: PLoS Negl Trop Dis. 2026 Apr 13;20(4):e0014187. doi: 10.1371/journal.pntd.0014187 (PMC13108900; doi:10.1371/journal.pntd.0014187)
Supplement: S1 Table — (PDF) [file pntd.0014187.s001.pdf]

Table S1. Summary of literature search for the typical area scanned by flyers. The citation number is linked to the main manuscript.

Citation	Mosquito	Measure/keywords	Estimation
	Culex spp.		
[26]	*Culex neavei*	Linear regression,	1356 m (Fig. 3)
	Culex poicilipes	maximum distance	843 m (Fig. 3)
[27]	*Cx. quinquefasciatus*	Isotype enrichment, dispersal,	59% $1-2$ km from natal larval habitat
		seeking oviposition	15% $>$ 2 km (Fig. 10B)
			26% $<$ 1 km (Fig. 10B)
[28]	*Cx. Pipiens*	Mean distance travelled (MDT)	Total, min: 0.16 km, max: 1.98 km
	Cx. salinarius		MDT: 1.33 km (Table 1)
[32]	*Culex* spp. genera	Average flight distance,	Avg. 609.5 m (SD 437.0 m) (Table 4)
		average maximum distance	Max. 5014 m (Table 3)
	Aedes spp.		
[26]	*Ae. vexans*	Linear regression,	1394 m (Fig. 3)
		maximum distance	
[27]	*Ae. albopictus*	Host seeking	100% $<$ 1 km
			79% $<$ 0.25 km
[29]	*Ae. lineatopennis*	Mean distance travelled	0.15 km (Table 2)
		post emergence	
[30]	*Ae. aegypti*	Mean distance travelled,	106 m (95% CI: 87.68, 123.69)
		weighted mean distance	(Fig. 2)
[31]	*Ae. aegypti*	Oviposition traps, mean	50% $<$ 32 m
		dispersal distance,	mean 45.2 m (95% CI: 39.7, 51.3)
		Laplacian kernel	10% $>$ 100 m
[32]	*Aedes* spp. genera	Average flight distance,	Avg. 89.0 m (SD 50.1 m)
		average maximum distance	Max. 2959 m (Table 3)
