# Supplementary figures and images for: Sensitivity analysis of factors influencing the ecology of mosquitoes involved in the transmission of Rift Valley fever virus
Source: PLoS Negl Trop Dis. 2026 Apr 13;20(4):e0014187. doi: 10.1371/journal.pntd.0014187 (PMC13108900; doi:10.1371/journal.pntd.0014187)

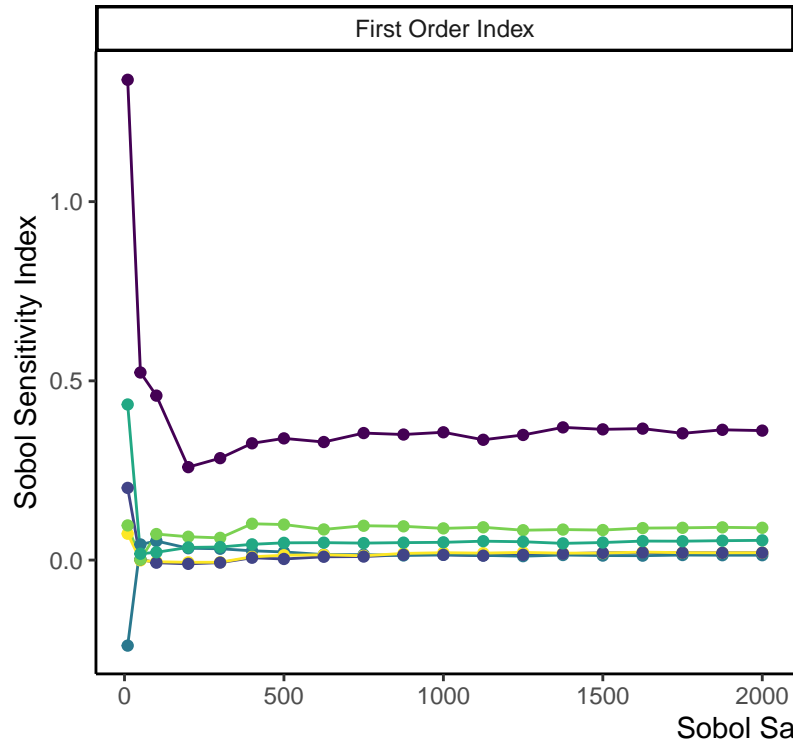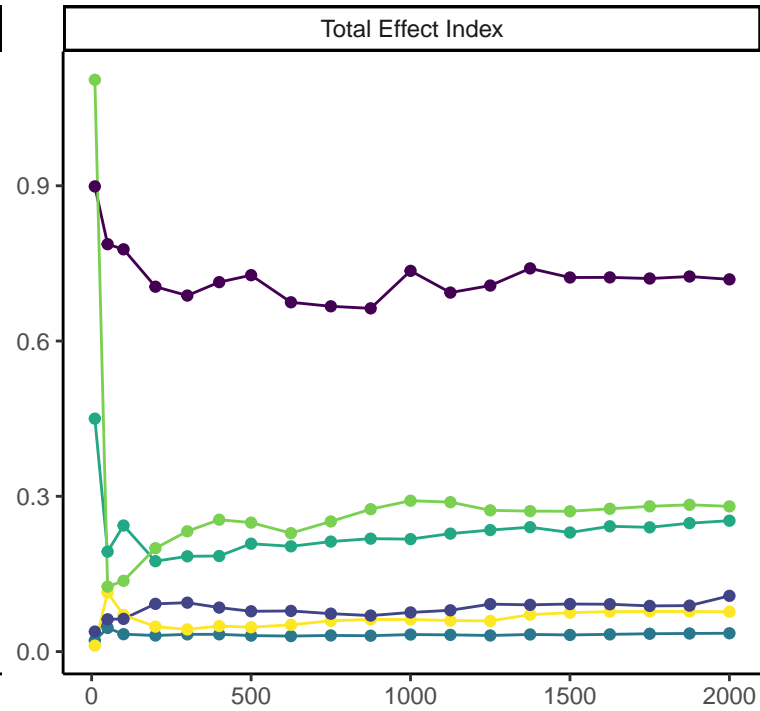

Supplement: S1 Fig — Evaluating the robustness of the Sobol sensitivity indices with increasing sample sizes. (PDF) [file pntd.0014187.s002.pdf]

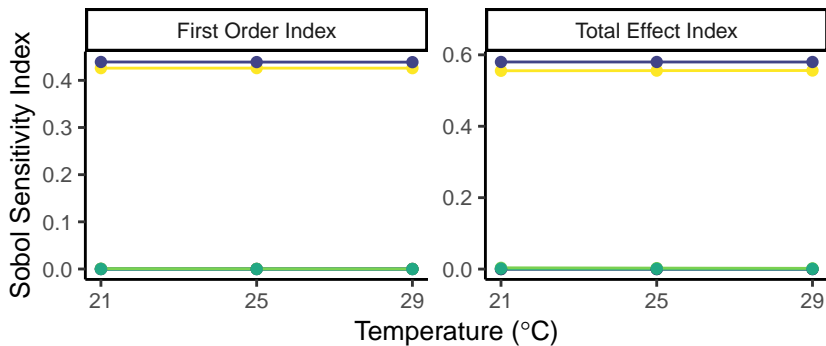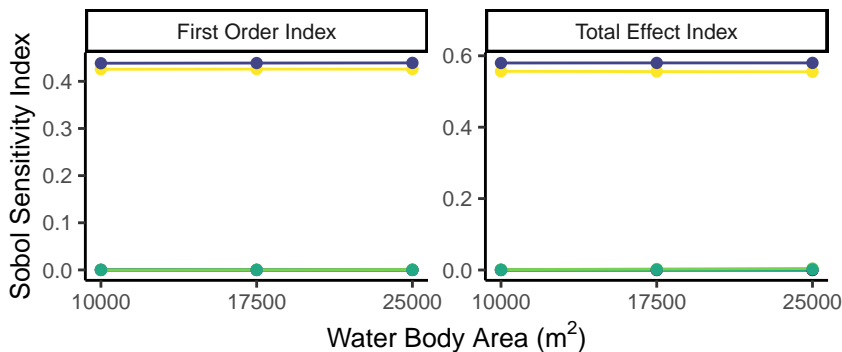

### Parameters

- $A_A$
- $\kappa^{Aedes}$
- $b_A$
- Livestock Total
- $q$
- $\rho_A$

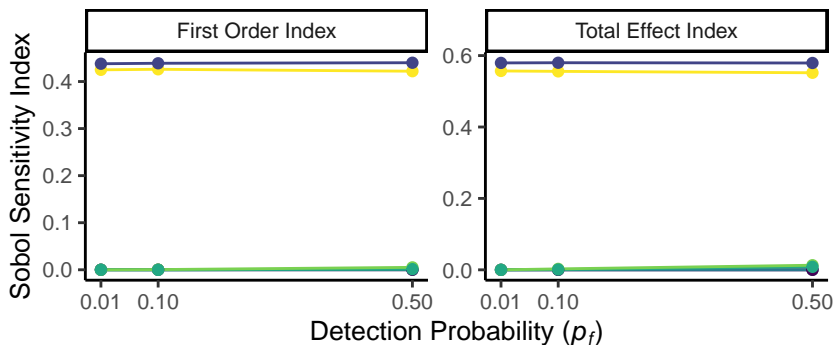

Supplement: S2 Fig — (PDF) [file pntd.0014187.s003.pdf]
