# Supplementary material for: Sensitivity analysis of factors influencing the ecology of mosquitoes involved in the transmission of Rift Valley fever virus
Source: PLoS Negl Trop Dis. 2026 Apr 13;20(4):e0014187. doi: 10.1371/journal.pntd.0014187 (PMC13108900; doi:10.1371/journal.pntd.0014187)
Supplement: S3 Fig — (PDF) [file pntd.0014187.s004.pdf]

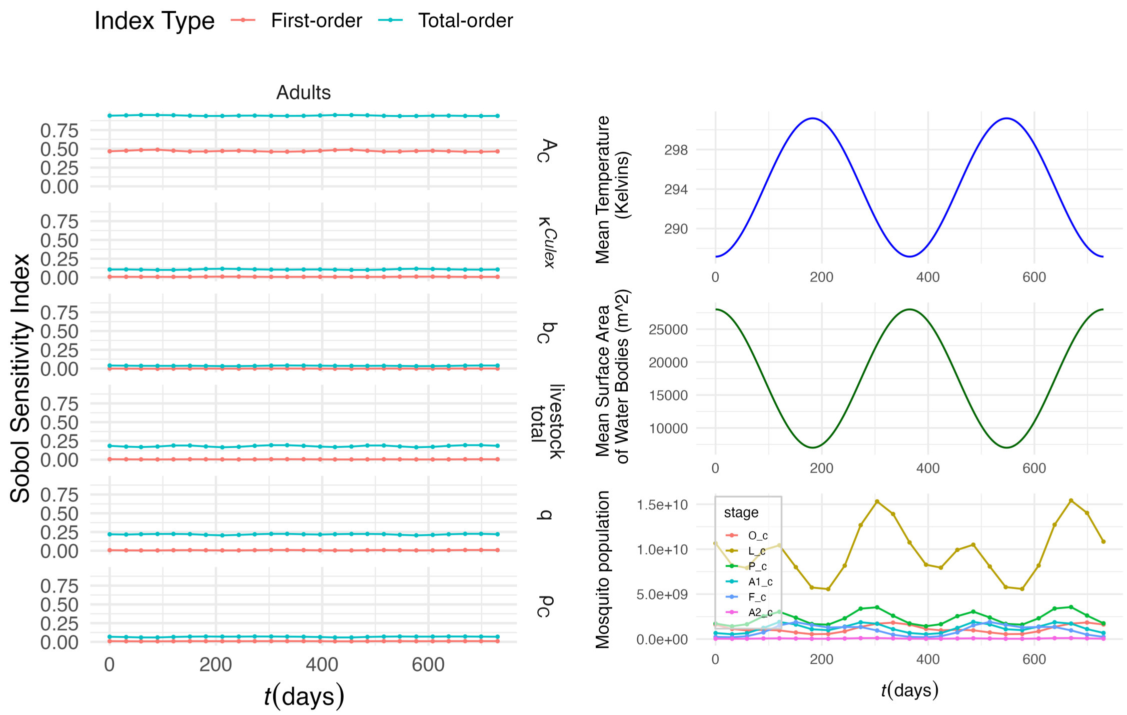

(a) *Culex*

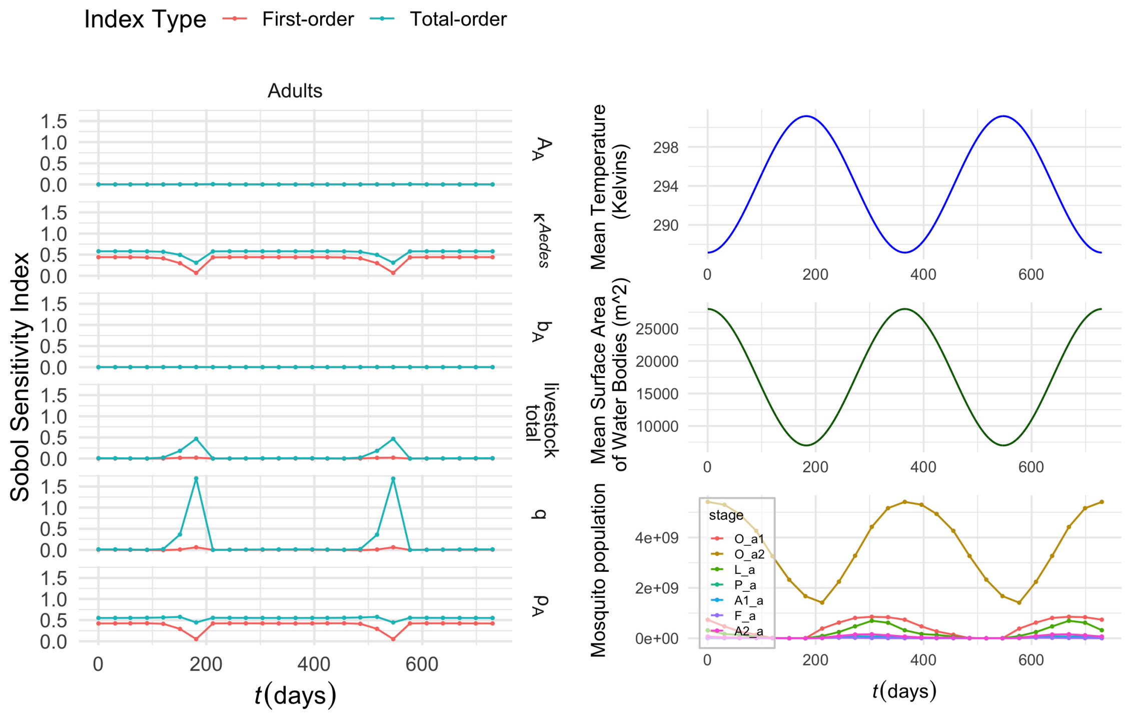

(b) *Aedes*

Fig S3. Time varying Sobol sensitivity indices for *Culex* and *Aedes* with out-phase periodic functions for temperature and water bodies.
