# Supplementary material for: Sensitivity analysis of factors influencing the ecology of mosquitoes involved in the transmission of Rift Valley fever virus
Source: PLoS Negl Trop Dis. 2026 Apr 13;20(4):e0014187. doi: 10.1371/journal.pntd.0014187 (PMC13108900; doi:10.1371/journal.pntd.0014187)
Supplement: S1 Appendix — (PDF) [file pntd.0014187.s005.pdf]

S1 Appendix

Calculation of distributions

Typical area scanned by *Aedes* flyers

Mosquito dispersal distances are often right-skewed, with most individuals travelling short distances while a few travel much farther. To model this distribution, a Gamma distribution has been chosen, as it is well-suited for positively skewed data and has been used in ecological dispersal studies [1]. The Gamma distribution is parameterized by a shape parameter (k) and a scale parameter (θ), which determine the distribution's form. These parameters are estimated using the known mean dispersal distance (μ) and an assumed variance (σ^2), following the relationships,

$$\mu = k\theta, \quad \sigma^2 = k\theta^2. \tag{1}$$

Given an estimated mean radius distance of 89 m (based on the broadest dataset [2]), the overall expected dispersal distance was calculated as $\mu = \pi \times 89^2$. Dispersal studies have indicated that the distribution is not highly skewed, but it does have a long tail. A lower k (e.g. $k < 1$) would result in a heavily skewed distribution, which may overestimate the proportion of mosquitoes travelling very short distances. On the other hand, a higher k (e.g., $k > 5$) would produce a more symmetric distribution, which may underestimate long-range dispersers. Hence, here we chose $k = 2.5$ to allow for a peak at a common dispersal distance while still maintaining a long right tail. Using this mean and an appropriate choice of k, the scale parameter θ was derived to ensure consistency with empirical data.

Number of eggs per batch

We propose that the number of eggs per batch follows a normal distribution. This assumption is supported by several empirical studies cited that report egg counts across different mosquito species and experimental conditions as mean ± standard error or within defined ranges. These distributions generally exhibit symmetric variation around a central mean (e.g., 90 ± 12 eggs in *Aedes aegypti* [3]), with no evidence of heavy skew or zero-inflation. While biological counts are non-negative and sometimes skewed, the observed ranges (e.g., 60–125 eggs) do not strongly suggest departure from normality in this case. Hence, in the absence of raw individual-level data, and given the moderate spread and central tendency in reported means and standard deviations, the normal distribution provides a reasonable and tractable approximation for modeling fecundity.

To estimate the parameters of this normal distribution, specifically the mean (μ) and standard deviation (σ), we combine the available data from the various studies. To estimate the overall mean (μ_{overall}), we calculate the arithmetic mean of the means reported in each study. This provides a central estimate of the number of eggs per batch across all studies, and is computed as

$$\mu_{\text{overall}} = \frac{\sum_{i=1}^{n} \overline{X_i}}{n}, \tag{2}$$

where $\overline{X_i}$ is the mean from study i and n is the number of studies. If a mean is provided as a range, we use the midpoint of the range as the best estimate for $\overline{X_i}$.

To estimate the overall standard deviation (σ_{overall}), we account for the variability in the means across the studies. The overall standard deviation is estimated by computing the root-mean-square of the standard deviations (or standard errors) from the studies, which captures the spread of all the data across all studies. The formula for the overall standard deviation is,

$$\sigma_{\text{overall}} = \sqrt{\frac{\sum_{i=1}^{n} \sigma_i^2}{n}}, \quad (3)$$

where σ_i is the standard deviation reported in study i. This approach provides a reasonable estimate of the spread of the data across the studies.

References

1. de Freitas Costa E, Schneider SI, Carlotto GB, Cabalheiro T, de Oliveira Júnior MR. Zero-inflated-censored Weibull and gamma regression models to estimate wild boar population dispersal distance. Japanese Journal of Statistics and Data Science. 2021; p. 1–23. doi:10.1007/s42081-021-00124-0.

2. Verdonschot PFM, Besse-Lototskaya AA. Flight distance of mosquitoes (Culicidae): A metadata analysis to support the management of barrier zones around rewetted and newly constructed wetlands. Limnologica. 2014;45:69–79. doi:10.1016/j.limno.2013.11.002.

3. Yan J, Kibech R, Stone CM. Differential effects of larval and adult nutrition on female survival, fecundity, and size of the yellow fever mosquito, *Aedes aegypti*. Frontiers in Zoology. 2021;18(1). doi:10.1186/S12983-021-00395-Z.
