# Supplementary material for: Sensitivity analysis of factors influencing the ecology of mosquitoes involved in the transmission of Rift Valley fever virus
Source: PLoS Negl Trop Dis. 2026 Apr 13;20(4):e0014187. doi: 10.1371/journal.pntd.0014187 (PMC13108900; doi:10.1371/journal.pntd.0014187)
Supplement: S2 Appendix — Input parameters for the model. (PDF) [file pntd.0014187.s006.pdf]

S2 Appendix

Parameters

The values for each parameter that have been used throughout the stability analysis, which can be found in the supplementary material of [1]. For ease and reproducibility, they have been presented in Table S2.

environment if table fits in text column.

Table S2. Parameter values used when running the code.

Parameter	Symbol in Code	Value
Change for Stability Analysis		
Number of days in simulation	day_length	$365 * 11$
Constant Temperature (Table 1)	constanttemp	25
Constant Water Body Area (Table 1)	constantwb	17 500
Livestock Total	livestocktotal	500
Detection probability, p_f (Table 1)	prop_find	0.1
Remain for Total Analysis		
Time Step	delta_t	1
Number of ponds in each cell	N_ponds	1
Reduction rate for *Culex*, based on Figure 8 in [2]	reduction_C	0.5
Reduction rate for *Aedes*, based on Figure 8 in [2]	reduction_A	0.58
Birth rate for livestock	b_L1	1/(5 * 365)
Death rate for livestock	mu_L1	1/(5 * 365)
Parameter for the impact of the livestock on vector fecundity and gonotrophic cycles	q_divided	1.00E+11
Probability of transovarial transmission	q_A	0.007
Latent Period	epsilon_L1	2/7
Infectious Period	gamma_L1	1/30
Average time for egg deposition	t_dep	0.229
Parameters for Periodic Functions		
Frequencies of oscillations in surface areas of water bodies	omega_S_p	2*3.14/(365)
Frequencies of oscillations in surface areas of temperature	omega_T_a	2*3.14/(365)
Mean surface area of water bodies during periods $2\pi/\omega_S$	C_S_p	17500
Mean temperature during periods $2\pi/\omega_T$	C_T_a	21
The maximum amplitudes in the water oscillations	A_S_p	(1-0.4)*C_S_p
The maximum amplitudes in the temperature oscillations	A_T_a	7
Respective water phases	phi_S_p	acos(-1)†
Respective temperature phases	phi_T_a	acos(-1)†

†Values for phases are equal to this when the periodic functions are in phase (i.e. reach a peak at the same time). We use phi_T_a = acos(0) for out of phase.

Note that the daily larva mortality and daily pupa mortality for *Culex* are defined, respectively, as

$$\mu_L^{Culex} = 37.9317808331 - 0.2573339304 \cdot T + 0.0004364566 \cdot T^2 \quad (1)$$

$$\mu_P^{Culex} = 80.3113158804 - 0.5439116495 \cdot T + 0.0009210259 \cdot T^2, \quad (2)$$

where T is the temperature measured in Kelvins. Similarly, the daily larva and pupa mortality for *Aedes* are defined, respectively, as

$$\mu_L^{Aedes} = 50.1205 - 0.3393650263 \cdot T + 0.0005747698 \cdot T^2 \quad (3)$$

$$\mu_P^{Aedes} = 3.524873 - 0.023943082 \cdot T + 0.00004066735 \cdot T^2. \quad (4)$$

If the rounded figures are used from the supplementary material [1], then a negative death rate occurs. These are defined as stated here in the code.

References

1. Lo Iacono G, Cunningham AA, Bett B, Grace D, Redding DW, Wood JLN. Environmental limits of Rift Valley fever revealed using ecoepidemiological mechanistic models. Proceedings of the National Academy of Sciences of the United States of America. 2018;115(31):E7448–E7456. doi:10.1073/pnas.1803264115.

2. Reisen WK, Fang Y, Martinez VM. Effects of temperature on the transmission of West Nile Virus by *Culex tarsalis* (Diptera: Culicidae). Journal of Medical Entomology. 2006;43(2):309–317. doi:10.1093/jmedent/43.2.309.
