# Supplementary material for: Sensitivity analysis of factors influencing the ecology of mosquitoes involved in the transmission of Rift Valley fever virus
Source: PLoS Negl Trop Dis. 2026 Apr 13;20(4):e0014187. doi: 10.1371/journal.pntd.0014187 (PMC13108900; doi:10.1371/journal.pntd.0014187)
Supplement: S5 Appendix — Using the same Sobol matrices, we put them in the opposite mosquito system to observe any differences. (PDF) [file pntd.0014187.s009.pdf]

S5 Appendix

Investigation into differences

The Sobol matrices are based on the distributions observed in Table 3. The minimum and maximum values for each parameter can be seen in Table S3, along with the range and the percentage deviation from the midpoint. A higher percentage indicates a wider spread of values relative to the midpoint. If the percentage is low, it means the range is small compared to the midpoint, implying less variability.

Table S3. Parameter range in Sobol Matrix for $N = 1500$ samples.

Parameter	Minimum	Maximum	Range	Percent
$\mathcal{A}_C$	464 866	15 593 548	15 128 682	188%
$\mathcal{A}_A$	783	102 848	102 065	197%
κ^{Culex}	0.001 48	0.9985	0.997 02	199%
κ^{Aedes}	0.001 48	0.9985	0.997 02	199%
b_C	95.6	202.3	106.7	72%
b_A	45.7	64.2	18.5	34%
ρ_C	519 043	19 990 479	19 471 436	190%
ρ_A	20 957	999 521	978 564	192%

Notably, the parameter representing the number of eggs laid per batch has the smallest percentage range. Combining insights from the literature review and this sensitivity analysis, it becomes evident that this parameter is the least influential compared to the three other parameters under investigation. This suggests that selecting a value within its given range results in minimal impact on the overall outcomes.

To examine the impacts of the parameter ranges on the models, we ran the sensitivity analysis within the *Culex* model with the *Aedes* parameters and the *Aedes* model with the *Culex* parameters (Fig S9). It can be observed that the order of significance of the Sobol indices have remained the same, with minimal differences between the size of the index.

Thus, the consistency in the order of Sobol indices across models, despite the swapped parameter ranges, indicates that the observed differences are primarily driven by the extent of parameter variability rather than any structural or functional issues within the models themselves.

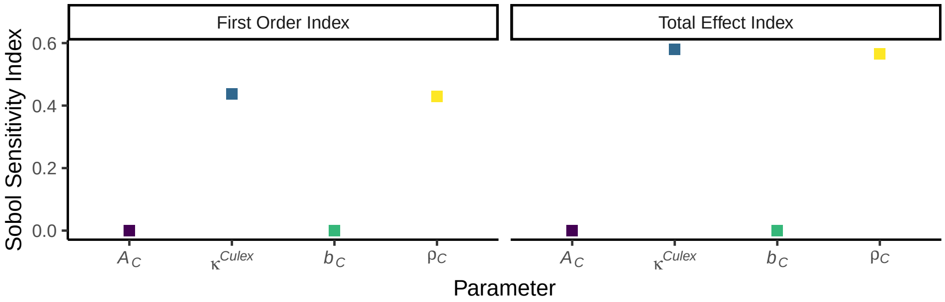

A

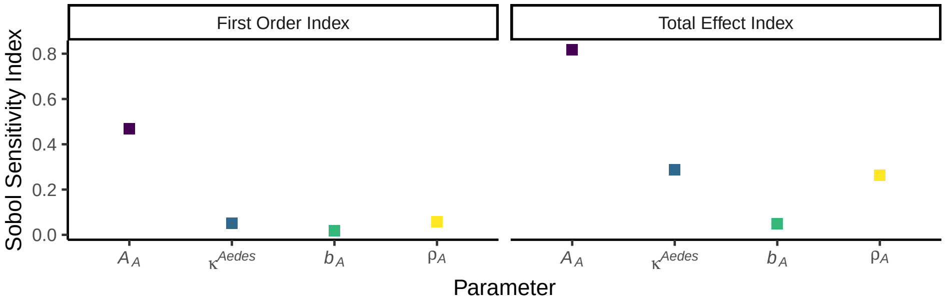

B

Fig S9. First order and total effect Sobol sensitivity indices with medium values of temperature, water body area and detection probability. A: *Culex* mosquitoes using the *Aedes* parameter ranges for the sensitive parameters. B: *Aedes* mosquitoes using the *Culex* parameter ranges for the sensitive parameters.
