# Supplementary material for: Sensitivity analysis of factors influencing the ecology of mosquitoes involved in the transmission of Rift Valley fever virus
Source: PLoS Negl Trop Dis. 2026 Apr 13;20(4):e0014187. doi: 10.1371/journal.pntd.0014187 (PMC13108900; doi:10.1371/journal.pntd.0014187)
Supplement: Attachment — Submitted filename: sensitivity analysis review.pdf [file pntd.0014187.s010.pdf]

The manuscript entitled *“Sensitivity analysis of factors influencing the ecology of mosquitoes involved in the transmission of Rift Valley Fever Virus”* presents a deterministic compartmental model to evaluate the sensitivity of ecological parameters that shape mosquito ecology. By applying Sobol global sensitivity analysis and literature-based parameter ranges, the authors identify influential parameters and highlight essential knowledge gaps, particularly regarding oviposition site use. This approach is methodologically sound and relevant, as it advances understanding of the ecological drivers underlying Rift Valley fever transmission dynamics. However, while the Sobol global sensitivity analysis provides useful insights into the relative importance of ecological parameters, the current approach treats sensitivity as static across the entire simulation horizon. Given that mosquito ecology and RVFV transmission risk are highly seasonal and time-dependent, I strongly recommend that the authors consider conducting a **time-dependent (dynamic) sensitivity analysis**. This would allow them to capture temporal shifts in parameter influence (e.g., rainfall dominating early in the season vs. oviposition later), which is crucial for both ecological understanding and practical vector control planning.

If full implementation is not feasible within the current submission, at a minimum the authors should discuss the limitations of a static approach and outline how time-dependent sensitivity analysis could improve the robustness of future modeling work.

Here are minor issues that the authors should address to clarify specific methodological points:

1) The abstract does not clearly articulate the public health implications of the findings. As written, it gives the impression that the contribution is primarily methodological.

2) The literature review focuses exclusively on *Aedes aegypti*. The authors must justify this choice, whether it is due to the relevance of this species in the Kenyan context or a lack of species-specific data for other vectors.

3) The rationale for selecting the four specific parameters over other potential model parameters needs further elaboration. The authors explain that these four are the most uncertain, but is that alone sufficient to justify excluding other parameters that may also influence the ecology?

4) Justify the choice of parameter distribution (uniform distribution) for \kappa and \rho. Is this biologically defensible?

5) Discuss the convergence of the Sobol’s indices.

6) Quantifying and discussing this interaction (e.g., does the importance of egg density depend on the proportion of area available?) would provide much richer ecological insight. This can be undertaken by exploring other orders.

7) The constant vs. periodic environment scenarios are mentioned, but their comparative outputs are not deeply analyzed (e.g., how periodic changes alter sensitivity ranks over time).

8) The discussion does not leverage key results with essential implications (e.g. the differential sensitivity of *Culex* and *Aedes* to water body size should be discussed in the context of habitat targeting for control)

9) The outcomes from the sensitivity analysis imply that factors affecting vector fecundity are less critical than habitat structure?

10) Host detection is not a limiting factor in the current model. The authors should discuss whether this holds under the condition of low livestock density.
