# Supplementary material for: Sensitivity analysis of factors influencing the ecology of mosquitoes involved in the transmission of Rift Valley fever virus
Source: PLoS Negl Trop Dis. 2026 Apr 13;20(4):e0014187. doi: 10.1371/journal.pntd.0014187 (PMC13108900; doi:10.1371/journal.pntd.0014187)
Supplement: Attachment — Submitted filename: Response to Reviewers.pdf [file pntd.0014187.s011.pdf]

PNTD-D-25-01142
Sensitivity analysis of factors influencing the ecology of mosquitoes involved in the transmission of Rift Valley fever virus
PLOS Neglected Tropical Diseases

Many thanks to the editor and reviewers for the constructive comments. We agree with the comments, and we have revised the manuscript accordingly. Here we consider each comment in turn, with the line numbers relating to the tracked-changed document.

Methods

Reviewer #1:

1. **The methods are appropriately described and the sensitivity analysis is interesting.**

We thank the reviewer for the supportive comment.

2. **In the discussion of what studies were included in the literature review I would have appreciated more detail on why things were included or excluded at each stage. For instance what in the titles made you retain or cut a study? Etc.**

Thank you for this comment, we have now added the Pubmed search strategy as well as extra details in the manuscript (lines 80-84). For example, in response to one reviewer's feedback (see comment 12 below) we clarify that we excluded studies referring to oviposition deterrence or attraction involving chemicals etc were excluded. The justification is that investigating how livestock-derived chemical plumes influence mosquito oviposition would require an interdisciplinary approach, incorporating not only mosquito biology but also the physics of turbulent flow, specifically, how chemical plumes (e.g., CO_2 emitted by a sheep) disperse and meander in the environment. We have now included a note on this in the discussion section. We acknowledge that this is an important and underexplored area of research.

Reviewer #2:

Introduction

3. **Line 18: I suggest removing the phrasing that Pepin "coined" the terms, as this was not the first mention. It is fine to cite Pepin et al., but not as the primary origin.**

Noted and removed.

Methods

4. **I appreciate the decision to simplify the model by removing some layers of complexity. This makes the analysis easier to interpret, but it would be worth noting in the discussion that RVF-endemic landscapes are highly heterogeneous, and simplification may limit applicability.**

We agree with the reviewer, and we have now added a section in the discussion on the limitations of our model as well as suggestions for future research (lines 831-838).

5. **The description of the Sobol methodology appears sound. While I am not a technical expert on Sobol indices, the explanation and approach are appropriate for the aims of the study.**

We thank the reviewer for the supportive comment. In response to reviewers' comments we have now extended the analysis to include time-varying Sobol analysis for the periodic case, with the advantage of seeing the importance of the parameters as the system changes over time. Additionally, we have expanded the methods section to provide more detail on the approach for calculating Sobol indices, aiming to make it clearer for readers without a technical background in this area. We have also explored a higher-order index as part of the analysis.

6. **Please clarify your consideration of oviposition behavior: eggs are rarely laid in the middle of large water bodies. How was this handled in the model assumptions?**

We agree with the reviewer. We assumed that *Culex* mosquitoes lay their eggs in the inner area around the edge of the water body (see Fig. S14 in the Supplementary Information of paper [15]). However, the model does not explicitly account for the spatial geometry of water bodies; instead, this inner area is represented as a proportion of the total water body area, denoted by κ^{Culex}. Similarly, we assumed that *Aedes* spp. lay their eggs in the moist soil surrounding the water body, and this moist soil area is likewise represented as a proportion of the total water body area, denoted by κ^{Aedes}. Both κ^{Culex} and κ^{Aedes} are among the parameters explored in this study, as there are limited data available on their values. We now clarify this in lines 106-132.

Reviewer #3:

7. **This work contains impressive modeling and sensitivity analysis of the models.**

We thank the reviewer for the supportive comment.

8. **I am not skilled in evaluating this kind of model nor this kind of sensitive analysis. However I do have substantial background in mosquito ecology and the epidemiology of Rift Valley fever virus.**
   There are assumptions in two of the authors' key parameters (oviposition density and area scanned for oviposition sites) that are in my opinion not correct.
   For oviposition density for both species it is not appropriate and potentially highly misleading to only consider surface area of the water body.
   This is because the perimeter (i.e., total length) of the water's edge is the biologically relevant factor.
   A perfectly circular pond and a crenellated (star shaped) edge pond could have the exact same water area but have vastly different perimeters.

We understand the issue raised by the reviewer. Let's start with a perfect circle, we argue that it is not the **perimeter** but rather the **annulus** (the ring-shaped area between two concentric circles, representing the zone surrounding the edge of a water body where egg laying occurs), that is biologically relevant. Let the **radial thickness** (i.e., the difference between the outer and inner radii) be denoted by Δr. The area of the annulus is then:

$$\pi(r + \Delta r)^2 - \pi r^2 = \pi(\Delta r^2 + 2r\Delta r).$$

This quantity is neither the total area nor the perimeter of the water body. If Δr had always the same value irrespective of the size of the water body, then the area of the annulus increases with the perimeter at a constant rate. We think, however, it is more realistic to assume that $\Delta r = \alpha r$, meaning that the annulus thickness is proportional to the inner radius (i.e., for *Aedes* larger water bodies have a wider zone of moist soil around the edge). Substituting this relationship gives an annulus area of $\pi(\alpha^2 + 2\alpha)r^2$. In this case, $(\alpha^2 + 2\alpha)$ corresponds to κ^{Aedes}. We agree however, that the true Δr is a complex function of multiple factors, including the area and shape (and therefore the perimeter) of the water body, as well as environmental variables such as temperature and evaporation rate. We discuss this in the revised manuscript (lines 105-127). In response to the reviewer's comment, we have also discussed the situation based on the perimeter. Let's assume that the relevant parameter is the perimeter. In this case we need to redefine the term ρ_A as the maximum linear density of eggs rather than superficial, *i.e.*, we assume that mosquitoes lay their eggs on a narrow line which can be approximated as a unidimensional line rather than surface. This ensures that correct dimensional analysis and the term $\rho_A\kappa^{Aedes}$ is still a number of eggs. Since we have no estimates of the linear density ρ_A , we prefer to leave this as future analysis. We have however, highlighted the issue and the way to solve this (lines 127-132). Similar argument for *Culex*.

Furthermore, it is interesting to note that it is possible for certain shapes to have a finite area but an infinite perimeter (e.g., the Koch snowflake). Although this may seem paradoxical, it illustrates the risk

of assuming that the number of mosquitoes eggs is proportional to the perimeter: a water body with a highly fragmented border could have an extremely large perimeter, leading to an unrealistic estimate of egg numbers.

9. **Perimeters are where the eggs are laid and so perimeters determine how many eggs are there.**
   Also, as a population, Aedes mosquitoes produce and oviposit multiple batches of eggs and continue to oviposit as the water recedes, right at the water's edge.
   Thus there could be multiple concentric perimeters of eggs at any given water body.

This effect is intrinsically included in the model, except that we are considering annuli rather than the perimeter (see above). For example, as shown in equation (2), over time, the area of the water bodies (and therefore their perimeters, which are proportional to the square root of the area) shrink and expand cyclically. Consequently, at each time step, the region where *Aedes* lay their eggs changes continuously, forming multiple concentric annuli of eggs around each water body. The effect on sensitivity analysis is now explicitly captured through the use of time-varying Sobol analysis.

10. **Also most of the parameterization in this model comes from Aedes Stegomyia (aegypti and albopictus) - neither are species currently of nigh importance in RVFV epidemiology in endemic areas.**

Regarding the fact that most parameters come from *Aedes albopictus* and *Aedes aegypti*, this highlights the paucity of data for non-model species. Note that we did not voluntarily restrict the data to those species as shown by the literature review search methodology. There are also data related to other species. As we have said in the discussion and conclusion of our manuscript, we have addressed this point by conducting a sensitivity analysis to quantify the influence of these parameters.

11. **Regarding scanning for oviposition sites (based on dispersal distance) - most of those studies from which the authors draw parameters were measuring dispersal of host-seeking females (newly emerged), not gravid blood fed females looking for oviposition sites.**
    There is no reason to believe that host seeking females would not return to the same water body they came from after blood feeding nearby.

Again, this point highlights the paucity of data on the distance travelled by females looking for oviposition sites. However, some data are directly related to females seeking oviposition sites (as seen for *Culex* and *Aedes* in ref [27]).

As we mentioned in the introduction of our manuscript, our findings highlight a significant knowledge gap surrounding spatial patterns of mosquito oviposition across larger bodies of water. Many dispersal estimates in the literature derive from mark–release–recapture studies of host-seeking or newly-emerged adults rather than gravid, oviposition-seeking females (Guerra 2014; Marini et al. 2019) and their measurements are also biased. Because gravid females may display skip-oviposition or site-fidelity depending on species and habitat availability, we treat dispersal as uncertain and have adopted a conservative approach.

12. **Perhaps a more important parameter is distance to livestock host and the degree of ephemerality of the water body where immature development took place.**

We acknowledge the importance of the ephemerality of the water body where immature mosquito development occurs. In response to the reviewer's comment, we have now incorporated this factor into the **time-varying sensitivity analysis** (see lines 209-215 for method description and lines 539-572 for results).

The model is not spatially explicit. Developing a model that accounts for the distance between livestock and mosquito breeding sites would constitute an entire research programme in itself. As mentioned above and in the discussion section, such an approach would also require investigating how livestock-derived chemical plumes influence mosquito oviposition. This would involve not only mosquito biology

but also the physics of turbulent flow, specifically how chemical plumes disperse and meander in the environment. We have now added a note on this in the discussion section (lines 89-95). However, the distance to livestock hosts is implicitly represented in the model through their density (i.e., the number of livestock within the study area), under the assumption that livestock are uniformly distributed across the area. In the revised manuscript, livestock density is an additional parameter of investigation for the sensitivity analysis (see lines 460-467).

13. **Finally, the authors really need to be quite explicit on how this work is going to inform future RVFV epidemiology research and operational relevance.**

We explicitly provide in the revised manuscript which parameters are in most need to be measured accurately, the public health implications and future research (see sections “Implication for public health and control strategies”, “Limitations and future research directions”)

14. **I am generally frustrated by RVFV modeling because the assumptions and parameterization are frequently fatally flawed - so please forgive my pointed criticism of this study.**

We fully share the reviewer’s concern. In our view, a key limitation, not only for RVFV epidemiology but for many vector-borne diseases, is the limited integration of high-quality interdisciplinary and transdisciplinary research that combines mathematical modelling, ecological and epidemiological fieldwork, and engagement with affected communities. Such an approach is essential to ensure that models accurately reflect ecological realities and retain practical relevance. We now highlight this point in the Discussion section, lines xxx.

15. **Again, the modeling and mathematical capabilities of the authors might be beyond reproach, but in my view the biological realism is not as good as it could be and is likely misleading the modeling effort which could have real consequences if it is taken up into public health policy.**
    RVFV epidemiology in endemic areas is diverse:
    The 2-species (Aedes/Culex) model only accounts for the major decadal epizootics and is really an East African phenomenon and seems to be less and less relevant compared to the burgeoning inter-epizootic nature of the virus throughout the potential transmission area in endemic lands.
    Epidemiology of the virus in Southern Africa, Madagascar, Egypt, Arabian Peninsula, Western and Central Sahel — all very different, and especially to the dambo system in East Africa.

We understand the frustration of the reviewer, this is a limitation for any biological or mathematical model. As said above, to address these limitations, we need a programme that combines modelling and fieldwork, with each component informing the other to improve accuracy and realism. The present study represents a first step towards model-guided fieldwork, by identifying the parameters that are likely to have the greatest influence on the model outputs. In the Discussion, we highlight the need for initiatives that specifically support the integration of modelling and fieldwork. We also discuss the epidemiological idiosyncrasies in different geographic settings (lines 839-843)

16. **I urge the authors to target their modeling to advocate for mass larviciding in the mambo system and gather parameters and intervention modeling specific to this end.**

The current sensitivity analysis is not equipped to adequately assess the impact of mass larviciding. However, the model can be extended to include additional ecological factors such as competition among larvae, predation effects, and vegetation dynamics. These extensions would enable a more thorough evaluation of larvicide application and its optimal timing. We have briefly addressed this important aspect in lines 789-792 and 878-882

Reviewer #4:

17. **The description of model structure and parameters is not sufficiently detailed. Readers unfamiliar with the earlier model might struggle to follow how mosquito dynamics are simulated.**

In response to the reviewer's comment, we now provide a high-level description of the model and its structure. However, due to space limitations, readers are referred to the supplementary work of Lo Iacono et al. (2018) for the complete mathematical formulation.

18. **Parameter ranges and distributions used in the sensitivity analysis are not always clearly justified (why those ranges? based on literature, expert opinion, or assumptions?).**

The choice of parameter ranges and distributions is based on the literature, with references listed in Table 2. In cases where information on the range or distribution was unavailable, we used a uniform distribution, which avoids bias in the selection of values and represents a state of total ignorance within the chosen range. This is clarified in lines 368-372.

19. **The paper does not fully explain how Sobol indices were computed, which may limit reproducibility for readers outside the field.**

Given the length of the paper, we have tried to minimise the amount of detail on how they were computed. A link to the github repository is provided in the text (reference 25). Here the full details of the code are available to reproduce the results. We have added some further explanation to the text, along with references to the packages and used in R.

20. **The Sobol indices are presented as aggregated across time, but mosquito ecology is inherently time-varying. This means the results miss important dynamics (e.g., rainfall may dominate early season, oviposition later). A time-dependent sensitivity analysis (Sobol indices as a function of time) would add much more ecological realism.**

In the first analysis (figs 1-3), we assumed constant temperatures and water body availability (not rainfall). Consequently, after a transient phase driven by random initial conditions, the mosquito population reached equilibrium, and time-varying Sobol indices were not required. We agree with the reviewer that, under periodically varying temperatures and water bodies, the mosquito population becomes strongly time-dependent; therefore, as suggested by the reviewer, we applied time-varying Sobol sensitivity analysis in this case, as now discussed in 209-215.

The use of water bodies, as in Lo Iacono et al. (2018) and in the present study, reflects their suitability as a direct proxy (without delay) for breeding sites. In contrast, when using rainfall, a delay must be incorporated to account for the time required for breeding site formation and subsequent oviposition. This is now discussed in lines 858-862.

21. **The methods do not specify whether empirical calibration/validation of parameters was done using field data (e.g., mosquito counts, oviposition studies). Without at least partial validation, the model risks being too theoretical.**

A direct empirical calibration or validation of the model parameters was not possible due to the absence of suitable field data. However, the model was indirectly and partially validated by comparing the

simulated distribution of inter-epidemic periods for Rift Valley fever with empirical data (Figure 4 in Lo Iacono et al. 2018), using observed water-body and temperature data.

A full parameter calibration would require high-resolution longitudinal data on mosquito abundance (ideally across life stages), together with locally measured air temperature and water-body dynamics. Such data would allow inference of unknown parameters using approaches such as Bayesian methods; however, given the large number of parameters, this is extremely challenging.

The focus of this work is therefore to identify the most influential parameters and those with high uncertainty, which warrant further attention. Parameter distribution information can also inform priors for future Bayesian inference and help prioritize influential parameters when high dimensionality limits inference (lines 56-59).

We also highlight a key limitation, common to RVFV and many vector-borne diseases: the lack of integrated, high-quality interdisciplinary research combining mathematical modeling, ecological and epidemiological fieldwork, and engagement with affected communities. Such integration is essential to ensure models reflect ecological realities and retain practical relevance. This point has now been emphasized in the Discussion section.

Results

Reviewer #1:

22. **The results are easy to follow, and the figures are nicely done. Currently the results for the sensitivity analysis are separated by culex and aedes but the discussion merges the two, as does the rest of the paper. It would have made more sense to me to separate these by parameters again and discuss both mosquito species at the same time under each parameter.**

We have opted to merge the discussion / conclusion for *Aedes* and *Culex* rather than splitting them by species. This decision was made to avoid unnecessary repetition and to facilitate a more straightforward comparison between the two species. We hope this approach meets the reviewer's expectations.

Reviewer #2:

23. **The results are well presented, with clear figures that effectively support the findings.**

We thank the reviewer for the supportive comment. Please note, in response to reviewer comments we have added some extra figures for the time-varying sensitivity analysis in the main text and in supplementary material.

Reviewer #3:

24. **See above - problem with parameters**

Please see our response highlighted above.

Reviewer #4:

25. **The objectives state the use of sensitivity analysis on ecological factors but do not clearly define all the parameters under investigation from the start (focus seems to emerge later on oviposition). The framing is broad ("factors influencing mosquito ecology") but narrows abruptly to four parameters, which could confuse readers.**

We now list the chosen parameters in the Introduction and clarify the rationale for their selection. Specifically, the sensitivity analysis was conducted only on independent parameters whose values are

not derived from other quantities. In contrast, parameters such as the vector-to-host ratio and developmental rates, although potentially sensitive, are defined by mechanistic relationships with temperature, water-body availability, and host abundance, and are therefore not subject to arbitrary variation (lines 46-57).

26. **While RVFV transmission is the context, the objectives do not clearly connect the ecological modeling to potential applications in vector control or outbreak preparedness. The results highlight that oviposition proportion is the most influential factor, but quantitative detail is limited.**

In response to the reviewer's comment, we have added a dedicated section in the Discussion titled "Implications for public health and control strategies", see also new section "Limitations and future research directions"

27. **Sobol indices can capture interaction effects, but the manuscript mainly emphasizes main effects, leaving out valuable insights about parameter synergies.**

We thank the reviewer for this comment.

The total-effect Sobol index provides an estimate of the overall influence of a parameter, including both its direct (main) effect and all interactions (synergies) it engages in with other parameters. While these indices do not resolve the contributions of specific parameter pairs or groups, they do comprehensively reflect each parameter's total impact, both alone and through interactions, on model output variance. We have now included a second-order index analysis for the medium constant scenario, with results shown in appendix 4, and discussed in line 692-716.

28. **The constant vs. periodic environment scenarios are mentioned, but their comparative outputs are not deeply analyzed (e.g., how periodic changes alter sensitivity ranks over time).**

We agree with the reviewer, and we now use time-varying Sobol indices under periodically varying temperatures and water bodies, see comment 27 above. This is now discussed throughout section 'Identifying influential parameters'.

Conclusions

Reviewer #1:

29. **The conclusions seem fair. I found it interesting the the important parameters differed for the two mosquito species but discussion of this was scant. If the authors wish they could expand this part of the discussion for biologists.**

In response to the reviewer's comment, we have added a dedicated section in the Discussion titled *"Implications for public health and control strategies."*

Reviewer #2:

Discussion

30. **Line 413: Please clarify the statement on disease spread via vector dispersal, noting that infected livestock movement was not considered in the model.**

We now have added in that although the model is capable of simulating the disease, we focused on the ecological component only.

31. **If a major goal of the study is to guide future data collection; please be more explicit about which field data are most needed to refine the model and which factors should be prioritized in future sampling. Your paper is likely to have more (or just as many) field epidemiologist readers than modellers.**

We have added a dedicated section in the Discussion titled “Gaps in data collections” and “Limitations and further research directions”

32. **Line 430: Given the absence of oviposition data across water bodies, is it reasonable to assume the entire surface area is available for egg laying? Some comment on this assumption would be helpful.**

Please see discussion in 105-132 and response to reviewers above (Comments 8 and 9).

33. **Line 482: Since mosquitos must feed on a host in order to produce eggs, is it acceptable to ignore this in the model? This should be acknowledged as a limitation.**

This important aspect is actually included in the model, using the parameterization of Pelosse et al (2013) for triatomines (reference 17), please see discussion in 844-847.

34. **Please add a few lines discussing the overlap between ecological factors influencing oviposition and host availability, and whether this missing linkage constrains the model’s realism.**

Again, please see above and discussion in 830-838.

35. **I suggest adding a comment that this model could be useful for vector control programs targeting reductions in multiple mosquito species, not just Aedes spp. All need to lay eggs and feed**

We have amended the manuscript accordingly lines 749-752

Reviewer #3:

36. **See above - problem with parameters**

Please see our response highlighted above.

Reviewer #4:

37. **While the dominance of oviposition is valid, the discussion underplays the role of other parameters (temperature, water body fluctuations) and how they interact in real-world ecological systems. The paper calls for more field data but does not sufficiently engage with existing entomological studies on oviposition ecology in RVFV vectors.**

The effects of temperature and water body fluctuations are intrinsically incorporated into the model, specifically impacting the oviposition rate, egg load rate, development time of newly laid *Aedes* eggs, and their hatching rate (see equations 3-5, 8-9, 15 and 19 in the supplementary materials of Lo Iacono et al. 2018). Temperature is also included in the mortality rates of eggs and the development rates of mosquito stages. The time varying Sobol analysis now incorporates time-varying temperatures and water body conditions.

38. **The discussion does not strongly connect the findings to disease surveillance or control strategies (e.g., how monitoring water bodies could inform early-warning systems). The contribution of the study (e.g., being one of the first to apply global**

sensitivity analysis to RVFV mosquito ecology) could be highlighted more clearly to strengthen the impact.

We thank the reviewer for the suggestion. We have added a dedicated section in the Discussion titled "*Implication for public health and control strategies*"

Editorial and Data Presentation Modifications?

Reviewer #1:

39. **I would present the 4 parameters you compared, in two species (so really 8 parameters) in the abstract/intro and not just vaguely say 4 parameters. I had to read all the way through the results to find what parameters for mosquitos you were going to use in the sensitivity analysis. Also it was not clear to me until the results that you did 4 parameters in two species of mosquitoes - and the results differed by species so consider clarifying this in the abstract.**

We agree with the reviewer about the lack of details in the abstract. We have addressed this now.

Reviewer #2: (No Response)

Reviewer #3:

40. **Comma after i.e. and e.g.**

We have made sure there is a comma after both.

41. **Check italicization of species names.**

We have gone through and checked all and italicized those that were missed.

Reviewer #4:

42. **The manuscript entitled "Sensitivity analysis of factors influencing the ecology of mosquitoes involved in the transmission of Rift Valley Fever Virus" presents a deterministic compartmental model to evaluate the sensitivity of ecological parameters that shape mosquito ecology. By applying Sobol global sensitivity analysis and literature-based parameter ranges, the authors identify influential parameters and highlight essential knowledge gaps, particularly regarding oviposition site use. This approach is methodologically sound and relevant, as it advances understanding of the ecological drivers underlying Rift Valley fever transmission dynamics.**

    However, while the Sobol global sensitivity analysis provides useful insights into the relative importance of ecological parameters, the current approach treats sensitivity as static across the entire simulation horizon. Given that mosquito ecology and RVFV transmission risk are highly seasonal and time-dependent, I strongly recommend that the authors consider conducting a time-dependent (dynamic) sensitivity analysis. This would allow them to capture temporal shifts in parameter influence (e.g., rainfall dominating early in the season vs. oviposition later), which is crucial for both ecological understanding and practical vector control planning.
    If full implementation is not feasible within the current submission, at a minimum the authors should discuss the limitations of a static approach and outline how time-dependent sensitivity analysis could improve the robustness of future modeling work.

We thank the reviewer for the constructive criticism. We have now performed a time-varying sensitivity analysis, and we also discuss further analyses (e.g., using time-varying environmental factors with different frequencies and phases) to improve the robustness of future modelling work.

43. **Here are minor issues that the authors should address to clarify specific methodological points:**
    a. **The abstract does not clearly articulate the public health implications of the findings. As written, it gives the impression that the contribution is primarily methodological.**

The abstract has been adjusted to make it clearer what parameters we are analysing, as mentioned above, and a sentence has been added at the end of the abstract to indicate that there are public health implications of the findings.

b. **The literature review focuses exclusively on Aedes aegypti. The authors must justify this choice, whether it is due to the relevance of this species in the Kenyan context or a lack of species-specific data for other vectors.**

It is a lack of species-specific data for other vectors. We now clarify this in the revised manuscript lines 598-600.

c. **The rationale for selecting the four specific parameters over other potential model parameters needs further elaboration. The authors explain that these four are the most uncertain, but is that alone sufficient to justify excluding other parameters that may also influence the ecology?**

We now list the chosen parameters in the introduction and clarify the rationale for their selection. Specifically, the sensitivity analysis was conducted only on independent parameters whose values are not derived from other quantities. In contrast, parameters such as the vector-to-host ratio and developmental rates, although potentially sensitive, are defined by specific mechanistic relationships with temperature, water availability, and host abundance, and are therefore not subject to arbitrary variation (lines 51-59).

d. **Justify the choice of parameter distribution (uniform distribution) for \kappa and \rho. Is this biologically defensible?**

We clarify the rationale for using a uniform distribution, which avoids bias in the selection of values and represents a state of total ignorance within the chosen range. This is a common choice in such contexts; for example, uniform priors are frequently used in Bayesian inference when no strong prior information is available.

e. **Discuss the convergence of the Sobol's indices**

We have now added in discussion about the convergence of the Sobol's indices (lines 239-251), and Figure S1 to accompany the analysis. With the additional parameters we are including in the analysis, we have decided to increase the number of Sobol runs to N=2000 instead of N=1500.

f. **Quantifying and discussing this interaction (e.g., does the importance of egg density depend on the proportion of area available?) would provide much richer ecological insight. This can be undertaken by exploring other orders.**

Thank you for this comment. We have extended the analysis to now include second order indices in the results with the method discussed in the supporting information. We decided not to include any higher order terms as the indices were small so no useful information could be gained from them.

g. **The constant vs. periodic environment scenarios are mentioned, but their comparative outputs are not deeply analyzed (e.g., how periodic changes alter sensitivity ranks over time).**

In response to reviewer comment, we have now performed a time-varying Sobol analysis in the periodic environment lines 539-572.

h. **The discussion does not leverage key results with essential implications (e.g. the differential sensitivity of Culex and Aedes to water body size should be discussed in the context of habitat targeting for control)**

We now discuss this point in the Discussion in the "*Implication for public health and control strategies*" section.

i. **The outcomes from the sensitivity analysis imply that factors affecting vector fecundity are less critical than habitat structure?**

Not necessarily. Sensitivity analysis not only identifies the important and influential parameters that drive model outputs and their magnitudes, but it also determines the extent to which uncertainty in structural model parameters contributes to the overall variability in the model output. Therefore, a large index may simply reflect high uncertainty in the input parameter. This uncertainty is epistemic in nature, as it arises from limited field data, and could be reduced through targeted data collection. We have now clarified this point in the manuscript and included recommendations for future fieldwork to reduce such uncertainty.

j. **Host detection is not a limiting factor in the current model. The authors should discuss whether this holds under the condition of low livestock density.**

The impact of livestock number was already included in the model. However, in response to the reviewer's comment, we have now incorporated it into the time-varying and constant sensitivity analysis for two additional parameters: the number of livestock and the parameter representing its effect on vector fecundity and gonotrophic cycles (denoted by q in Table S3 of Lo Iacono et al., 2018). The findings are fully discussed in the Discussion section.

Summary and General Comments

Reviewer #1:

44. **This manuscript is interesting and useful to folks modelling vector-borne disease - specifically RVF. I would have appreciated some discussion about whether the authors think the parameters that are important in the sensitivity analysis would hold in other similar vector-borne diseases or if they think this is RVF specific?**

We now clarify that although the approach is based on a model developed for RVFV transmission, its findings are relevant to other mosquito species with oviposition behaviours similar to those of *Aedes* and *Culex* spp., which exemplify egg-laying on water and on moist soil, respectively (lines 749-752).

Reviewer #2:

45. **This manuscript presents a sensitivity analysis of ecological factors influencing mosquito dynamics relevant to RVFV transmission. Using a deterministic, compartmental model developed for RVFV in Kenya, the authors examine the relative influence of parameters such as oviposition area, egg density, and dispersal on mosquito abundance.**
    The study is well presented and addresses an important gap in understanding how environmental conditions shape vector populations. The figures are clear and the methodological approach, while simplified, is accessible and informative. The main strength is the identification of oviposition area as a highly influential parameter, highlighting the need for targeted field data. I would suggest the authors to think about what this might mean for future vector control programmes and how they think this model fits within the broader field since it has been submitted to a multidisciplinary journals and readers will ask "what does this mean, practically?"

We recommend more research on oviposition in the conclusion and discussion section as gaining data on the ecology of vectors of RVF could help design better strategies for mosquito control and disease prevention (see sections “Gaps in data collections“, “Implication for public health and control strategies” and “Limitations and future research directions”). Please, note that our model did not incorporate any epidemiological component as an output and that it might be premature to give strict recommendations in term of disease control.

Key overall points:

46. **Please use spp. when referring to mosquito genera.**

Done.

47. **Emphasize in the limitations that vertical transmission and hatching of Aedes eggs is not the only way RVFV is maintained between outbreaks; livestock movements and secondary vectors (e.g., Culex, Mansonia) are also important. Why make this distinction if you combine them for the model? Your main point seems to be that both are important for transmission, but they have different life history traits -- which I would agree with**

We agree with the reviewer, in fact in Lo Iacono et al 2018 the model showed that RVFV could persist even without vertical transmission. We now discuss this point in the limitations (lines: 578-582).

48. **Consider discussing that breeding is not limited to water bodies alone; other oviposition sites matter, and water body size may also act as a proxy for rainfall variability.**

We now discuss this point in the limitations in lines 858-862.

49. **This is a very useful summary of mosquito parameters relevant for spatial analysis.**
50. **Ground some of the ecological assumptions more explicitly (e.g., does oviposition occur the middle of water bodies or mostly around the edge?).**

This is now explicitly discussed, as reviewed above (lines 105-132)

51. **I like that you say data collection needs to improve for parameterization, but strengthen the impact of the study by specifying exactly what new data are most needed to improve models like yours and which ecological factors should be prioritized in sampling approaches.**

We now discuss this point, please see sections “Gaps in data collections“ and “Limitations and future research directions”.

52. **While not required for publication, I do notice that no coauthors are from institutes in Kenya or East Africa. Such collaborations would be very useful to address some of the gaps around impact of the manuscript and implications for practical control programmes.**

Thank you for this comment regarding regional collaborations. We agree that partnerships with institutions in Kenya and across East Africa are important for ensuring that research findings have strong local relevance and applicability. This particular study was primarily methodological and modelling-focused, which was led by an interdisciplinary team including experts in mathematical ecology, and vector-borne disease dynamics.

Reviewer #3:

53. **Problem with parameters**

We agree with the reviewer that the potential applications to vector control and outbreak preparedness were not explicitly discussed in the original manuscript. One reason is that the study primarily aimed to identify parameters most in need of accurate measurement. It is important to emphasize, however, that this reflects either (i) their strong influence on mosquito ecology, thereby highlighting potential avenues for application, or (ii) their large uncertainty, which greatly affects model outputs and thus hampers the models' predictive power, or both (lines 753-764). The time-varying Sobol analysis, however, can provide important insights into when interventions targeting specific parameters are likely to have the greatest impact. We now discuss this in lines 728-742 and in the "Limitations and future research directions".
