# Supplementary material for: Sensitivity analysis of factors influencing the ecology of mosquitoes involved in the transmission of Rift Valley fever virus
Source: PLoS Negl Trop Dis. 2026 Apr 13;20(4):e0014187. doi: 10.1371/journal.pntd.0014187 (PMC13108900; doi:10.1371/journal.pntd.0014187)
Supplement: Attachment — Submitted filename: Response to ReviewersMarch2026.pdf [file pntd.0014187.s012.pdf]

We thank the reviewers for the kind comments and suggestions made to improve the manuscript prior to publication.

Reviewers' comments:
Reviewer's Responses to Questions

Key Review Criteria Required for Acceptance?

As you describe the new analyses required for acceptance, please consider the following:

Methods:
-Are the objectives of the study clearly articulated with a clear testable hypothesis stated?
-Is the study design appropriate to address the stated objectives?
-Is the population clearly described and appropriate for the hypothesis being tested?
-Is the sample size sufficient to ensure adequate power to address the hypothesis being tested?
-Were correct statistical analysis used to support conclusions?
-Are there concerns about ethical or regulatory requirements being met?

Reviewer #5*: Yes.*

Reviewer #6*: The methods used for the literature review were adequate. The model and parameters and sensitivity analysis methods were well explained for readers of different backgrounds.*

Reviewer #7*: Line 169: How do you define the typical area scanned by a mosquito before finding an oviposition site? I see that you define it later, but please elaborate further here to provide context.*
Added in a short elaboration:

... the typical area scanned by mosquitoes before finding an oviposition site, ideally measured from the movement of gravid females between blood-feeding and oviposition sites, but often inferred from general mosquito dispersal distances due to limited information ...

.

Reviewer #8*: (No Response)*

Results:
-Does the analysis presented match the analysis plan?

-Are the results clearly and completely presented?
-Are the figures (Tables, Images) of sufficient quality for clarity?

Reviewer #5*: Yes.*

Reviewer #6*: The results were linked to the aim and objective of the analysis. Referral was made to the mathematical model that was previously developed. The tables and figures were very informative and formatted according to best practices in writing standards.*

Reviewer #7*: Line 314: Add the caveat that Aedes albopictus are container/treehole mosquito species, not a flood pan/dambo species, so dispersal behaviour may be different. I would also reorganize this paragraph to list the Aedes spp. together and the Culex spp. together.*

We have added in the caveat about the species with an extra sentence:

"It should be noted that Aedes albopictus are container/treehole mosquito species, and not a flood pan/dambo species, so dispersal behavior may be different."

With regard to the ordering, the text was originally organised by publication rather than by species, to make it easier for readers to link directly to the relevant papers. Splitting solely by species is difficult because some publications refer to both *Aedes spp.* and *Culex spp*. However, we have now explicitly reorganised S1 Table in the Supporting Information by species and clearly refer to this in the manuscript with the following sentence:

"In the sections that follow, we organise our review by publication to facilitate direct linkage to the original studies. For readers who prefer the information grouped taxonomically, the same data are reorganised by mosquito species in S1 Table of the Supporting Information."

We hope the editor and reviewer are satisfied with this solution, and we are happy to move the table into the main text if they advise.

Line 371: Was egg production of wild and colonised-strains compared in this study? Or are you comparing to the study in line 369 of blood-deprived mosquitoes (which arguably is not a sound comparison of lab vs. field)? Please clarify.

References [35] and [36] are studies that were conducted under laboratory conditions. We included study [35] to represent extreme scenarios where females produce eggs without a blood meal because of their ability of autogeny. The range covered by the Sobol analysis was designed to encompass all potential scenarios, including such

extremes. We have explicitly noted this distinction in the manuscript at the point we introduce the references and further down when discussing the chosen mean and standard deviation for species.

Line 384: Again you should explicitly state the different ecologies of container breeders, like Ae. aegpyti and albopictus, and floodwater species like mcintoshi.
Added in the sentences below to state the differences between species:

Container-breeding species, such as Aedes aegypti and Aedes albopictus, typically lay eggs in artificial or natural containers such as water-filled tyres, pots, and tree holes, where water availability is relatively stable. In contrast, floodwater species like Aedes mcintoshi lay eggs in temporary water bodies formed by rainfall or flooding, which are highly transient and variable. These ecological differences influence their reproductive strategies and egg production patterns.

Line 381: Perhaps not particularly surprising - bloodmeals are more closely linked to fertility than sugar-feeding in mosquitoes.

Thank you for pointing this out. We have removed the 'surprising' and added this information at the end of the paragraph.

Line 396: What do you mean by nutritional status? Do you mean larval diet? Adult body mass? Bloodmeal volume/frequency? Please clarify.

The paper from Yan et al. refers to larval and adult diet for their nutritional status. These points have been added in for clarity:

Furthermore, Yan et al. \cite{yan_differential_2021} explored the effects of nutritional status, specifically larval and adult diet, such as nectar availability on fecundity. They reported that mosquito fecundity depends on both larval and adult nutrition: larval food quantity and adult food quality together determine egg production. Specifically, they found that well-nourished Aedes aegypti females laid an average of 90 eggs per batch, with a range of approximately 60 to 125 eggs

Reviewer #8: *(No Response)*

Conclusions:
-Are the conclusions supported by the data presented?
-Are the limitations of analysis clearly described?
-Do the authors discuss how these data can be helpful to advance our understanding of

the topic under study?
-Is public health relevance addressed?

***__Reviewer #5__**: Yes.*

***__Reviewer #6__**: Following the previous review, the authors made good conclusions with recommendations that specified gaps in data for model parameters.*

***__Reviewer #7__**: Line 577: Egg production may not be the best indicator of population size, because only a small proportion of these actually survive to adulthood. Please elaborate on the decision to focus on the egg stage. Also, you have not considered competition in this model – some mosquitoes avoid habitats that already have a high density of conspecific eggs or larvae.*

In this and our previous study, we focused on how water bodies (or rainfall) affect mosquito abundance and dynamics. Because water bodies determine the availability of oviposition sites, we explicitly included the egg stage in the model. The model incorporates egg mortality, so only a small proportion of eggs survive, as the reviewer notes. Predation is not modelled explicitly, although it could be incorporated into the mortality term. Density dependent competition is represented via a carrying capacity: when egg numbers are high, fertility is reduced. This is why egg density was included in the sensitivity analysis.

We now added the following sentence for clarification:

Some mosquitoes avoid habitats that already have a high density of conspecific eggs or larvae. To represent this density-dependent competition, the model included a carrying-capacity term based on egg surface density, which limits how many eggs a water body can support. As eggs become crowded, oviposition rates fall. For this reason, egg density was included in the sensitivity analysis.

Line 739: Although collecting more data is certainly a way of addressing uncertainty, it's worth acknowledging the challenges of directly collecting data for certain mosquito ecological/biological parameters (e.g. lifespan).

We have included a couple of sentences acknowledging this:

However, it is important to acknowledge the challenges of directly measuring some mosquito ecological and biological parameters, such as lifespan, due to practical and logistical constraints. In such cases, indirect estimation methods, modeling

approaches, or sensitivity analyses may provide valuable insights to address these data gaps.

***<u>Reviewer #8</u>**: (No Response)*

Editorial and Data Presentation Modifications?
Use this section for editorial suggestions as well as relatively minor modifications of existing data that would enhance clarity. If the only modifications needed are minor and/or editorial, you may wish to recommend "Minor Revision" or "Accept".

***<u>Reviewer #5</u>**: None.*

***<u>Reviewer #6</u>**: (No Response)*

***<u>Reviewer #7</u>**: Lines 8-9: I would argue that this isn't a modelling limitation so much as a data limitation (which the authors themselves point out in this paper). Consider rephrasing or adding that caveat to this sentence.*
Thank you, this has now been included:

However, existing models struggle to determine which parameters most influence vector dynamics due to limited data availability and model complexity.

Line 11: Is Garissa a state? Province? Indicate so that the scale of the outbreak is in context.

This has been addressed. It is the Garissa District.

Line 21: Please specify that these are floodwater Aedes spp.

This has been specified:

The former group is thought to primarily consist of floodwater Aedes spp. Mosquitoes …

Line 23: Their vector competence and role in transmission remains unclear, so I would change this to "may be involved".

This has been changed in text:

… though Mansonia and Anopheles may be involved as well.

Line 54: I don't quite understand what you mean by "are not subject to arbitrary variation". Pleased clarify this sentence.

We have changed this sentence and added a new sentence after to clarify that since the parameters are influenced by specific, predictable environmental or biological factors, then they are not influenced by random fluctuations.

These relationships constrain their variation, meaning that changes in these parameters are predictable and not random or arbitrary.

Please improve the resolution of all figures, particularly Figures 4 and 5, which are quite blurry.

We have improved the resolution – we had put the figures through the PLOS tool originally which limited the resolution. We have done this manually now to get the required resolution.

Reviewer #8*: (No Response)*

Summary and General Comments:
Use this section to provide overall comments, discuss strengths/weaknesses of the study, novelty, significance, general execution and scholarship. You may also include additional comments for the author, including concerns about dual publication, research ethics, or publication ethics. If requesting major revision, please articulate the new experiments that are needed.

Reviewer #5*: This manuscript presents a detailed deterministic compartmental model to evaluate the sensitivity of ecological parameters that shape the ecology of Culex and Aedes mosquitoes in the context of Rift Valley Fever Virus (RVFV) transmission. The authors clearly present their introduction, methods, results and discuss their findings, highlight current research gaps and list the limitations of the study. Furthermore, they have appropriately responded to the previous review comments. Although this manuscript is highly mathematical as it is model-based, they clearly present its findings in a way that can be used by people implementing RVFV prevention/control strategies and by researchers to fill up the existing gaps.*

Reviewer #6*: The authors conducted a sensitivity analysis of ecological input parameters that influence mosquito dynamics and, consequently, Rift Valley fever transmission patterns using a deterministic, compartmental model specifically designed for RVFV in Kenya. To determine the parameter values, they reviewed existing literature. This is a very useful study. The model focuses on ecology and mosquitoes, thereby contributing to understanding and predicting RVF outbreaks. However, because the RVF eco-epidemiology is very complex, involving a broad host system of livestock and wildlife, it has limitations. Following the review of the previous manuscript, the authors clearly defined and elaborated on these limitations. The model is also hindered*

by a lack of field data on certain input parameters, and they explain what further data collection is needed to better understand the value and distribution of these variables. The methods and results are well presented, and the text is generally clear, easy to follow, and flows smoothly. I think the authors did a very good job tackling this challenging analysis. This publication could significantly contribute to improving the prediction of RVF outbreaks when combined with findings from other studies focusing on factors such as horizontal transmission patterns and immunity variability in ruminant livestock and wildlife and historical data on animal and human cases.

Reviewer #7: *The authors present a variance-based sensitivity analysis of the ecological drivers of RVFV vector population dynamics. Although I am not overly familiar with Sobol analysis, I believe that the work provides valuable insights into the potential processes underpinning RVFV transmission. I have only a few comments that I would like to see addressed before the manuscript is published.*

We hope that these comments have been adequately addressed.

Reviewer #8: *(No Response)*
